# Exploring causal associations of alcohol with cardiovascular and metabolic risk factors in a Chinese population using Mendelian randomization analysis

**DOI:** 10.1038/srep14005

**Published:** 2015-09-14

**Authors:** Amy E. Taylor, Feng Lu, David Carslake, Zhibin Hu, Yun Qian, Sijun Liu, Jiaping Chen, Hongbing Shen, George Davey Smith

**Affiliations:** 1 MRC Integrative Epidemiology Unit (IEU) at the University of Bristol, Oakfield House, Oakfield Grove, Bristol, UK; 2 UK Centre for Tobacco and Alcohol Studies and School of Experimental Psychology, University of Bristol, Bristol, BS8 1TU, UK; 3 Department of Epidemiology & Biostatistics at Nanjing Medical University School of Public Health, 818 East Tianyuan Rd, Nanjing 211166, P.R. China; 4 Department of Chronic Non-communicable Disease Control, Zhejiang Provincial Center for Disease Control and Prevention, 3399 Binsheng Rd, Hangzhou 310053, P.R. China; 5 School of Social and Community Medicine, University of Bristol, Oakfield House, Oakfield Grove, Bristol, BS8 2BN, UK; 6 Department of Chronic Non-communicable Disease Control, Wuxi Center for Disease Control and Prevention, 499 Jincheng Rd, Wuxi 214023, P.R. China

## Abstract

Observational studies suggest that moderate alcohol consumption may be protective for cardiovascular disease, but results may be biased by confounding and reverse causality. Mendelian randomization, which uses genetic variants as proxies for exposures, can minimise these biases and therefore strengthen causal inference. Using a genetic variant in the *ALDH2* gene associated with alcohol consumption, rs671, we performed a Mendelian randomization analysis in 1,712 diabetes cases and 2,076 controls from Nantong, China. Analyses were performed using linear and logistic regression, stratified by sex and diabetes status. The A allele of rs671 was strongly associated with reduced odds of being an alcohol drinker in all groups, but prevalence of alcohol consumption amongst females was very low. The A allele was associated with reduced systolic and diastolic blood pressure and decreased total and HDL cholesterol in males. The A allele was also associated with decreased triglyceride levels, but only robustly in diabetic males. There was no strong evidence for associations between rs671 and any outcomes in females. Our results suggest that associations of alcohol consumption with blood pressure and HDL-cholesterol are causal. Alcohol also appeared to have adverse effects on triglyceride levels, although this may be restricted to diabetics.

Observational studies have provided evidence that moderate alcohol consumption may be beneficial for cardiovascular health^1^. A recent meta-analysis of 84 studies found lower risk of coronary heart disease and stroke incidence and mortality in drinkers compared to non-drinkers^2^. Intervention studies, including randomized trials, have also demonstrated that alcohol consumption is associated with increased high density lipoprotein (HDL) cholesterol, which is observationally protective for cardiovascular disease, but have not provided clear evidence for associations with other lipid fractions^3^.

Evidence emerging from Mendelian randomization studies indicates that some of these observed beneficial effects are probably due to residual confounding or reverse causality, and that drinking even small amounts of alcohol is unlikely to be beneficial for cardiovascular health^4^,^5^. In Mendelian randomization, genetic variants that are associated with alcohol drinking are used as proxies for measured alcohol consumption, which minimises biases from confounding and removes the possibility of reverse causality^6^,^7^. There is growing evidence from genetic studies that alcohol-related variants conferring higher alcohol consumption are associated with higher blood pressure and body mass index^4^,^5^,^8^,^9^,^10^, which are strong risk factors for cardiovascular disease. Several studies also provide support for the findings from intervention studies that alcohol consumption increases HDL cholesterol^11^,^12^. However, this finding has not been consistently replicated^5^. The role that alcohol consumption may have in determining triglyceride levels is less clear, with studies suggesting both positive and negative effects^5^,^8^,^9^,^12^.

In East Asian populations, the main genetic variant used in Mendelian randomization studies of alcohol use is located in the Aldehyde dehydrogenase 2 (*ALDH2*) gene, which is the major enzyme involved in the breakdown of acetaldehyde, a metabolite of alcohol. A single nucleotide polymorphism, rs671 (G > A) leads to an amino acid substitution from glutamate to lysine at residue 487, and the resultant subunit is non-functional^13^. Carriers of a single A allele have a reduced ability to clear acetaldehyde and tend to consume lower levels of alcohol, whilst individuals with two A alleles are unable to clear acetaldehyde and, consequently, many do not drink alcohol at all^14^. The associations of this variant with alcohol consumption have been widely replicated in Asian populations^4^. In Europeans, this variant is not polymorphic, so variants in other alcohol metabolizing genes e.g., alcohol dehydrogenases (*ADH*) genes are used in Mendelian randomization studies^8^. However, European studies require much larger sample sizes due to the smaller amount of variance explained in alcohol consumption by *ADH* variants.

Using data from a case control study of diabetes in China, we performed a Mendelian randomization study, using rs671, to further explore the causal role of alcohol in determining cardiovascular and metabolic traits.

## Methods

### Study population

The study population comprised diabetes cases and controls from an ongoing, population-based cohort study of about 40 000 subjects in Changzhou and Nantong in Jiangsu Province, China during 2004 and 2008. Details of this sample have been described previously^15^. Participants answered an interview-administered questionnaire and had anthropometric and cardiovascular measures taken. Methods were carried out in accordance with the approved guidelines. Written informed consent was obtained from every participant. This study was approved by the institutional review board of Nanjing Medical University (Nanjing, China).

### Cardiovascular measures

Height and weight were measured in light clothing, without shoes. BMI was calculated as weight(kg)/height(m)^2^.

Systolic blood pressure (SBP) and diastolic blood pressure (DBP) were measured after the subject had rested for at least 5 minutes. Two consecutive readings of blood pressure were taken on the right arm with a mercury sphygmomanometer according to 1999 World Health Organization/International Society of Hypertension guidelines^16^. An average of the two readings was used in the analysis.

Approximately 5 ml blood was collected from each participant after a 10-hour overnight fast. Fasting blood glucose (FBG), triglycerides, cholesterol and high-density lipoprotein (HDL) cholesterol were measured enzymatically (Hitachi 7180 Biochemistry Auto-analyzer, Japan) following the manufacturer’s instructions. Low density lipoprotein (LDL) cholesterol was estimated using the Friedewald equation. Individuals were classified as diabetic if they had a history of type 2 diabetes or if their FBG was ≥7.0 mmol L^−1^.

### Alcohol consumption

Self-reported alcohol consumption was categorised as a binary variable. Individuals were classified as drinkers if they reported drinking three or more alcoholic drinks a week, for more than six months at any point in their lifetime and non-drinkers if their alcohol consumption was lower than this.

### Genotyping

DNA was extracted from blood samples. The rs671 SNP was genotyped using the TaqMan assay on ABI PRISM 7900 HT platform (Applied Biosystems, Foster City, CA). Approximately equal numbers of case and control samples were assayed in each 384-well plate and two negative controls were used for quality control. Genotyping was performed by blinding the case or control status. The genotyping results were determined by using SDS 2.3 Allelic Discrimination Software (Applied Biosystems). The accordance rate was 100% for duplicates (5% of samples). There was no clear evidence for deviation from Hardy Weinberg Equilibrium in the total sample or amongst controls only (Pearson chi-square p-values ≥ 0.17).

### Statistical analysis

Analyses were conducted in Stata (version 13.1). Summary statistics were provided as means and standard deviations and medians and interquartile ranges (IQR) (for skewed variables), stratified by diabetes status and sex. The associations of rs671 genotype with alcohol drinking was analysed using logistic regression, stratified by diabetes status and sex. Associations of rs671 genotype with BMI, blood pressure, blood lipids and fasting blood glucose were assessed using linear regression, adjusted for age and stratified by sex. Sex stratification allows a test of the pleiotropy assumption because drinking prevalence is generally low in females in Asian populations and the rs671 variant will only affect outcomes through its influence on alcohol consumption in samples exposed to alcohol use. Therefore any effects of rs671 on outcome measures operating through differences in alcohol consumption would only be seen in males, whereas direct effects of the variant on outcome measures would be seen in both sexes^17^. Stratification by sex is preferable to stratifying by drinking status as it avoids the potential issue of collider bias^18^. Analyses were also performed additionally stratified by diabetes status to investigate if there were differences according to diabetes status. Heterogeneity between effect estimates by sex and diabetes status was tested using a likelihood ratio test to compare models with and without an interaction between the genotype and the stratifying variable. These models were adjusted for age, which was also allowed to interact with the stratifying variable. An additive genetic model was assumed for all analyses. Triglycerides and glucose were log transformed prior to analyses due to skewed distributions. Coefficients from these analyses were exponentiated and therefore represent ratios of geometric means; for these analyses the baseline value, indicating no association, is 1.

## Results

The total sample for analysis was 3,788 (614 diabetic males, 755 control males 1,098 diabetic females, and 1,321 control females) (see Table 1 for demographic characteristics). The mean age of the total study population was 57.6 years, but females tended to be older than males and diabetics older than controls (p < 0.001).

Prevalence of alcohol drinking was much higher amongst males (diabetics:44.5%, controls:52.3%) than females (diabetics:5.2%, controls:8.9%) and was slightly higher amongst controls than diabetic participants (p-values all <0.001). BMI, blood pressure, total cholesterol, triglycerides and fasting blood glucose were all higher and HDL cholesterol was lower in diabetics compared to controls (p-values all <0.001). There was also strong evidence for gender differences for all outcome measures apart from triglycerides and blood glucose in diabetics and LDL cholesterol in controls.

The minor (A) allele of rs671 was associated with reduced odds of being an alcohol drinker in all groups (diabetic males OR:0.20 (95% CI:0.14,0.28), control males: (OR:0.23, 95% CI:0.17,0.30), diabetic females (OR:0.26, 95% CI:0.13,0.52) control females (OR:0.23, 95% CI:0.14, 0.38)) (see supplementary Table S1). There was suggestive evidence that the minor allele of rs671 was associated with reduced odds of being diabetic in males (OR:0.85, 95% CI:0.71,1.01), but not in females (OR:1.00, 95% CI:0.88,1.15).

In males, each additional A allele of rs671, which is associated with lower alcohol consumption, was associated with 2.26 mmHg (95% CI:0.79,3.72) lower systolic blood pressure and 1.52 mmHg (95% CI:0.58, 2.45) lower diastolic blood pressure (Table 2). Each additional A allele was also associated with lower levels of total cholesterol (−0.12 mmol L^−1^, 95% CI:−0.21, −0.02) and HDL cholesterol (−0.05 mmol L^−1^, 95% CI: −0.09, −0.01) but not with LDL cholesterol in males. There was no strong evidence for heterogeneity by diabetes status for associations with these outcomes in males (p all ≥0.07; Supplementary Table S2), although beta coefficients were consistently stronger in diabetics. There was no clear evidence for association of rs671 with either BMI or fasting glucose in males.

There was also strong evidence in males that each A allele was associated with a reduction in triglyceride levels (ratio of geometric means:0.86, 95% CI:0.81, 0.92). However, when the sample was additionally stratified by diabetes, the effect was only observed in the diabetic males (0.79, 95% CI:0.71, 0.88) and was essentially null in the control males (0.97, 95% CI:0.92, 1.02) (p-value for heterogeneity <0.001) (see Supplementary material S2).

In females, there was no clear evidence to suggest that the rs671 SNP was associated with any of the outcomes assessed (Table 2). In addition there was little evidence for heterogeneity of associations by diabetes status within females (Supplementary Table S2). There was good evidence for heterogeneity between males and females in the associations between rs671 and blood pressure, total cholesterol and triglycerides (p all ≤0.03).

## Discussion

The minor allele of rs671, which is associated with lower prevalence of alcohol consumption, was associated with lower blood pressure, lower HDL cholesterol and lower triglycerides within the Nantong sample. These findings were observed in males but not females, who had a very low prevalence of alcohol drinking in this population, suggesting that the observed effect of rs671 on these risk factors operates through alcohol consumption.

Our findings which suggest alcohol consumption is related to higher blood pressure are consistent with those of previous Mendelian randomization studies in both Asian and European populations^4^,^8^,^11^,^19^. This study therefore contributes further to the evidence that alcohol consumption is likely to be detrimental to cardiovascular health in terms of raising blood pressure.

As reported here, the minor allele of rs671 has been found to be associated with lower levels of HDL cholesterol in several other studies^9^,^12^,^19^,^20^ which is in concordance with intervention studies linking alcohol drinking to higher HDL^3^. This finding should be interpreted with caution in terms of its suggestive beneficial effect of alcohol consumption on cardiovascular health. Whilst observationally higher levels of HDL are associated with reduced risk of coronary heart disease^21^, randomized controlled trials and Mendelian randomization studies using genetic variants associated with HDL provide evidence against HDL itself being a causal factor^22^,^23^,^24^,^25^.

We observed a negative association between the minor allele of rs671 and triglycerides, suggesting that alcohol consumption raises triglyceride levels, which would potentially have negative consequences for cardiovascular health^23^. The direction of this association is consistent with some previous studies of the effects of alcohol related variants on triglycerides^9^,^12^. However, this relationship is not well established in the literature. In a recent large meta-analysis of European individuals, an *ADH1B* variant which lowers alcohol consumption was associated with higher triglyceride levels^5^. It is also possible that the *ALDH2* rs671 variant may directly affect lipid levels, independently of alcohol consumption. In studies which have stratified analyses by drinking status, associations between *ALDH2* variants and some lipid fractions have been observed in samples of non-drinkers^26^,^27^. However, the lack of association with triglycerides in females in our study provides evidence against a direct association. It is unclear why the association with triglycerides was only seen in diabetic males within this sample or why associations with blood pressure and HDL cholesterol also tended to be stronger in diabetics. This could be the result of collider bias^28^, due to stratification on diabetes as we found some evidence that *ALDH2* genotype influences diabetes status.

This study would have been strengthened by more precise measurement of alcohol consumption, which could only be defined crudely using the self-reported questionnaire data. However, the association of this ALDH2 variant with alcohol consumption is widely reported in East Asian populations^4^,^11^,^29^,^30^ and the association of this variant with consumption in males but not females in our study is consistent with previous reports^4^.

## Conclusion

This study provides further evidence that the associations between alcohol consumption and increases in blood pressure and HDL cholesterol are causal. These data suggest that alcohol consumption may increase triglycerides, but this requires further investigation in other samples.

## Additional Information

**How to cite this article**: Taylor, A. E. *et al.* Exploring causal associations of alcohol with cardiovascular and metabolic risk factors in a Chinese population using Mendelian randomization analysis. *Sci. Rep.* **5**, 14005; doi: 10.1038/srep14005 (2015).

## Supplementary Material

Supplementary Information

## Figures and Tables

**Table 1:** Characteristics of the study population.

Variable	Males		P^*^	Females		P^*^
	Diabetic	Control		Diabetic	Control	
Age (years)	57.75 (10.61)	55.03 (11.69)	<0.001	59.35 (10.11)	57.59 (10.17)	<0.001
BMI (kg m^−2^)	24.88 (3.33)	21.56 (2.26)	<0.001	25.28 (3.67)	21.97 (2.53)	<0.001
SBP (mm Hg)	132.50 (19.26)	118.38 (12.65)	<0.001	134.84 (21.28)	116.86 (14.31)	<0.001
DBP (mm Hg)	81.80 (11.23)	75.14 (9.68)	<0.001	79.69 (11.17)	72.04 (9.10)	<0.001
Total cholesterol (mmol L^−1^)	4.58 (1.30)	4.15 (0.86)	<0.001	4.77 (1.19)	4.32 (0.79)	<0.001
HDL cholesterol (mmol L^−1^)	1.47 (0.53)	1.62 (0.38)	<0.001	1.54 (0.54)	1.71 (0.41)	<0.001
LDL cholesterol (mmol L^−1^)	2.19 (0.95)	2.12 (0.86)	0.19	2.33 (1.02)	2.16 (0.82)	<0.001
Triglycerides (mmol L^−1^)	1.67 (1.07, 3.18)	0.81 (0.62, 1.10)	<0.001	1.76 (1.20, 2.90)	0.93 (0.70, 1.21)	<0.001
Fasting blood glucose (mmol L^−1^)	8.01 (7.09, 10.35)	4.50 (4.10, 4.95)	<0.001	8.14 (7.09, 10.58)	4.56 (4.12, 5.00)	<0.001
% Drinkers	44.5	52.3	0.004	5.2	8.9	<0.001
Sample size range	514:614	745:755		955:1,098	1,311:1,321	

Means are shown with standard deviations and medians with inter-quartile ranges.

*P- value for difference between diabetics and controls from t-tests for continuous variables and chi-square exact tests for categorical variables.

**Table 2:** Associations of rs671 genotype with cardiovascular risk factors stratified by sex and adjusted for age.

Outcome	Sex	N	Linear regression			
			Estimate^*^	95% CI	P	P_het_†
BMI (kg m^−2^)	Female	2,397	−0.05	−0.28, 0.19	0.71	0.39
	Male	1,366	−0.21	−0.49, 0.07	0.14	
SBP (mm Hg)	Female	2,418	0.53	−0.75, 1.82	0.42	0.007
	Male	1,368	−2.26	−3.72, −0.79	0.002	
DBP (mm Hg)	Female	2,417	0.19	−0.53, 0.91	0.61	0.004
	Male	1,368	−1.52	−2.45, −0.58	0.001	
Total cholesterol (mmol L^−1^)	Female	2,410	0.01	−0.06, 0.08	0.73	0.03
	Male	1,367	−0.12	−0.21, −0.02	0.02	
HDL cholesterol (mmol L^−1^)	Female	2,411	−0.03	−0.06, 0.01	0.10	0.36
	Male	1,366	−0.05	−0.09, −0.01	0.01	
LDL cholesterol (mmol L^−1^)	Female	2,268	0.03	−0.04, 0.09	0.39	0.71
	Male	1,266	0.01	−0.07, 0.09	0.84	
Triglycerides (mmol L^−1^)^‡^	Female	2,411	1.02	0.98, 1.07	0.29	<0.001
	Male	1,365	0.86	0.81, 0.92	<0.001	
Fasting blood glucose (mmol L^−1^)^‡^	Female	2,410	1.01	0.99, 1.04	0.33	0.12
	Male	1,364	0.98	0.94, 1.01	0.22	

*Linear regression results represent difference in outcome per copy of A allele.

^†^P-value for heterogeneity between males and females from Likelihood Ratio Test.

^‡^Triglycerides and fasting blood glucose were log transformed. Beta coefficients represent ratios of geometric means, with 1 being the baseline value indicating no association.
