# Supplementary material for: Exploring causal associations of alcohol with cardiovascular and metabolic risk factors in a Chinese population using Mendelian randomization analysis
Source: Sci Rep. 2015 Sep 14;5:14005. doi: 10.1038/srep14005 (PMC4568464; doi:10.1038/srep14005)
Supplement: Supplementary Information [file srep14005-s1.doc]

**Supplementary material**

**Exploring causal associations of alcohol with cardiovascular and metabolic risk factors in a Chinese population using Mendelian randomization analysis**

Amy E.Taylor^1,2*^, Feng Lu^3,4*^, David Carslake^1,5^, Zhibin Hu^3^, Yun Qian^3,6^, Sijun Liu^3^, Jiaping Chen^3^, Hongbing Shen^3^, George Davey Smith^1,5^

1. MRC Integrative Epidemiology Unit (IEU) at the University of Bristol, Oakfield House, Oakfield Grove, Bristol, UK
2. UK Centre for Tobacco and Alcohol Studies and School of Experimental Psychology, University of Bristol, Bristol, BS8 1TU, UK
3. Department of Epidemiology & Biostatistics at Nanjing Medical University School of Public Health, 818 East Tianyuan Rd, Nanjing 211166, P.R. China
4. Department of Chronic Non-communicable Disease Control, Zhejiang Provincial Center for Disease Control and Prevention, 3399 Binsheng Rd, Hangzhou 310053, P.R. China
5. School of Social and Community Medicine, University of Bristol, Oakfield House, Oakfield Grove, Bristol, BS8 2BN, UK
6. Department of Chronic Non-communicable Disease Control, Wuxi Center for Disease Control and Prevention, 499 Jincheng Rd, Wuxi 214023, P.R. China

Table S1. Associations of rs671 genotype with alcohol drinking

|  | N | Drinkers' genotypes | | |  | Non-drinkers' genotypes | | |  | Logistic regression^1^ | | | |
| --- | --- | --- | --- | --- | --- | --- | --- | --- | --- | --- | --- | --- | --- |
| Stratum |  | GG | GA | AA |  | GG | GA | AA |  | OR | 95% CI | P | P_het_^2^ |
| Female Controls | 1311 | 100 | 16 | 1 |  | 654 | 479 | 61 |  | 0.23 | 0.14, 0.38 | <0.001 | 0.88 |
| Female Diabetics | 1089 | 48 | 9 | 0 |  | 589 | 377 | 66 |  | 0.26 | 0.13, 0.52 | <0.001 |  |
| Male Controls | 745 | 294 | 95 | 1 |  | 140 | 161 | 54 |  | 0.23 | 0.17, 0.30 | <0.001 |  |
| Male Diabetics | 607 | 225 | 42 | 3 |  | 152 | 155 | 30 |  | 0.20 | 0.14, 0.28 | <0.001 |  |

1. Age adjusted odds ratio for being a drinker per copy of the A allele for rs671
2. P-value for heterogeneity for difference between groups from Likelihood Ratio Test

Table S2. Associations of rs671 genotype with cardiovascular risk factors stratified by sex and diabetes status and adjusted for age

|  |  |  |  | Mean (SD) or Median^a^ (IQR) values among genotype: | | | Linear regression | | | | | |  |
| --- | --- | --- | --- | --- | --- | --- | --- | --- | --- | --- | --- | --- | --- |
| Outcome | Stratum | N |  | GG | GA | AA | |  | Estimate^1^ | 95% CI | P | P_het_^2^ | |
| BMI (kg m^-2^) | Female controls | 1317 |  | 21.96 (2.46) | 22.02 (2.64) | 21.75 (2.55) | |  | -0.01 | -0.24, 0.22 | 0.93 | 0.65 | |
|  | Female diabetics | 1080 |  | 25.39 (3.66) | 25.06 (3.75) | 25.53 (3.30) | |  | -0.10 | -0.46, 0.25 | 0.57 |  | |
|  | Male controls | 753 |  | 21.65 (2.34) | 21.44 (2.16) | 21.46 (2.11) | |  | -0.13 | -0.38, 0.12 | 0.31 | 0.57 | |
|  | Male diabetics | 613 |  | 24.91 (3.24) | 24.76 (3.47) | 25.26 (3.65) | |  | 0.01 | -0.43, 0.45 | 0.97 |  | |
| SBP (mm Hg) | Female controls | 1321 |  | 116.81 (14.23) | 117.26 (14.36) | 114.27 (14.83) | |  | -0.31 | -1.58, 0.96 | 0.63 | 0.09 | |
|  | Female diabetics | 1097 |  | 133.88 (21.12) | 136.11 (21.82) | 136.57 (19.43) | |  | 1.39 | -0.62, 3.40 | 0.18 |  | |
|  | Male controls | 754 |  | 118.71 (12.43) | 117.87 (12.67) | 118.13 (14.29) | |  | -0.61 | -2.04, 0.81 | 0.40 | 0.14 | |
|  | Male diabetics | 614 |  | 133.93 (20.44) | 130.42 (16.94) | 128.47 (16.86) | |  | -3.08 | -5.55, -0.62 | 0.01 |  | |
| DBP (mm Hg) | Female controls | 1320 |  | 71.98 (9.17) | 72.09 (8.96) | 72.34 (9.44) | |  | 0.14 | -0.69, 0.98 | 0.74 | 0.93 | |
|  | Female diabetics | 1097 |  | 79.74 (11.03) | 79.36 (11.39) | 81.14 (11.26) | |  | 0.23 | -0.85, 1.31 | 0.68 |  | |
|  | Male controls | 754 |  | 75.51 (9.60) | 74.69 (10.11) | 74.39 (8.07) | |  | -0.58 | -1.67, 0.50 | 0.29 | 0.09 | |
|  | Male diabetics | 614 |  | 82.59 (11.36) | 81.07 (10.99) | 77.09 (9.87) | |  | -2.13 | -3.60, -0.66 | 0.004 |  | |
| Total cholesterol (mmol L^-1^) | Female controls | 1313 |  | 4.33 (0.80) | 4.30 (0.77) | 4.30 (0.74) | |  | -0.03 | -0.10, 0.05 | 0.48 | 0.24 | |
|  | Female diabetics | 1097 |  | 4.73 (1.22) | 4.80 (1.16) | 4.86 (1.09) | |  | 0.06 | -0.06, 0.17 | 0.35 |  | |
|  | Male controls | 753 |  | 4.16 (0.86) | 4.16 (0.89) | 4.03 (0.67) | |  | -0.03 | -0.13, 0.07 | 0.53 | 0.08 | |
|  | Male diabetics | 614 |  | 4.66 (1.34) | 4.48 (1.27) | 4.23 (0.77) | |  | -0.20 | -0.37, -0.03 | 0.02 |  | |
| HDL cholesterol (mmol L^-1^) | Female controls | 1313 |  | 1.72 (0.45) | 1.71 (0.36) | 1.60 (0.31) | |  | -0.03 | -0.07, 0.01 | 0.14 | 0.92 | |
|  | Female diabetics | 1098 |  | 1.55 (0.56) | 1.52 (0.54) | 1.50 (0.38) | |  | -0.03 | -0.08, 0.03 | 0.34 |  | |
|  | Male controls | 752 |  | 1.63 (0.40) | 1.62 (0.37) | 1.51 (0.32) | |  | -0.04 | -0.09, 0.00 | 0.05 | 0.36 | |
|  | Male diabetics | 614 |  | 1.50 (0.54) | 1.43 (0.53) | 1.31 (0.45) | |  | -0.08 | -0.15, -0.01 | 0.03 |  | |
| LDL cholesterol (mmol L^-1^) | Female controls | 1313 |  | 2.16 (0.85) | 2.14 (0.78) | 2.22 (0.76) | |  | 0.00 | -0.07, 0.08 | 0.98 | 0.33 | |
|  | Female diabetics | 955 |  | 2.30 (1.02) | 2.36 (1.00) | 2.46 (1.07) | |  | 0.07 | -0.04, 0.17 | 0.23 |  | |
|  | Male controls | 752 |  | 2.11 (0.86) | 2.14 (0.89) | 2.14 (0.66) | |  | 0.03 | -0.07, 0.13 | 0.55 | 0.57 | |
|  | Male diabetics | 514 |  | 2.21 (1.00) | 2.12 (0.90) | 2.31 (0.82) | |  | -0.02 | -0.15, 0.12 | 0.80 |  | |
| Triglycerides^3^  (mmol L^-1^) | Female controls | 1313 |  | 0.95 (0.70, 1.21) | 0.90 (0.70, 1.20) | 1.05 (0.72, 1.31) | |  | 1.00 | 0.97, 1.04 | 0.99 | 0.20 | |
|  | Female diabetics | 1098 |  | 1.72 (1.18, 2.90) | 1.77 (1.23, 2.60) | 2.10 (1.41, 3.79) | |  | 1.05 | 0.98, 1.13 | 0.13 |  | |
|  | Male controls | 752 |  | 0.85 (0.64, 1.13) | 0.79 (0.60, 1.09) | 0.78 (0.68, 1.00) | |  | 0.97 | 0.92, 1.02 | 0.20 | <0.001 | |
|  | Male diabetics | 613 |  | 1.80 (1.16, 3.66) | 1.57 (1.02, 2.84) | 1.20 (0.90, 1.50) | |  | 0.79 | 0.71, 0.88 | <0.001 |  | |
| Fasting blood glucose^3^  (mmol L^-1^) | Female controls | 1312 |  | 4.53 (4.11, 5.00) | 4.58 (4.14, 5.00) | 4.60 (4.10, 5.08) | |  | 1.01 | 1.00, 1.02 | 0.26 | 0.43 | |
|  | Female diabetics | 1098 |  | 8.12 (7.02, 10.36) | 8.14 (7.10, 10.68) | 8.65 (7.21, 11.07) | |  | 1.02 | 0.99, 1.06 | 0.21 |  | |
|  | Male controls | 750 |  | 4.50 (4.13, 4.93) | 4.51 (4.05, 5.00) | 4.45 (4.00, 4.90) | |  | 0.99 | 0.98, 1.01 | 0.36 | 0.28 | |
|  | Male diabetics | 614 |  | 8.05 (7.11, 10.44) | 7.91 (7.00, 10.22) | 7.88 (7.20, 12.42) | |  | 1.02 | 0.97, 1.07 | 0.44 |  | |

1. Beta coefficients represent change in outcome per copy of the minor allele of rs671.
2. P-value for heterogeneity between controls and diabetics from Likelihood Ratio Test.
3. Outcome measures log transformed. Beta coefficients represent ratios of geometric means with 1 being the baseline value indicating no association.
